# Helminth species-specific effects on IFN-γ producing T cells during active and latent tuberculosis

**DOI:** 10.1371/journal.pntd.0011094

**Published:** 2023-01-20

**Authors:** Amare Kiflie, Gezahegn Bewket, Fitsumbrhan Tajebe, Ebba Abate, Thomas Schӧn, Robert Blomgran

**Affiliations:** 1 Department of Immunology and Molecular Biology, University of Gondar, Gondar, Ethiopia; 2 The Ohio State, Global One Health, Addis Ababa, Ethiopia; 3 Division of Inflammation and Infection, Department of Biomedical and Clinical Sciences, Faculty of Medicine and Health Sciences, Linkӧping University, Linkӧping, Sweden; 4 Department of Infectious Diseases, Kalmar County Hospital, Linkӧping University, Linkӧping, Sweden; 5 Department of Infectious Diseases, County of Östergötland, Linkӧping University Hospital, Linkӧping University, Linkӧping, Sweden; Consejo Nacional de Investigaciones Cientificas y Tecnicas, Fundación Mundo Sano, ARGENTINA

## Abstract

**Background:**

Interferon-γ (IFN-γ) is a key cytokine inducing protective immune responses during tuberculosis (TB) infection. Helminth-induced immune responses may affect IFN-γ production by T cells, although its connection with disease severity and immune recovery during treatment is unexplored. We investigated the species-specific effect of helminths on the IFN-γ production by T cells in relation to disease severity during active and latent TB infection (LTBI).

**Methods:**

In this study, 69 active pulmonary TB patients (PTB), 28 with LTBI and 66 healthy controls were included. Active TB was diagnosed using GenXpert MTB/RIF while QuantiFERON test (QFT) was used for the screening of healthy community controls (CCs) and for the diagnosis of LTBI. Helminth infection was identified by routine diagnosis whereas clinical disease severity was evaluated by the TB score. Intracellular IFN-γ production of T cells in stimulated peripheral blood mononuclear cells (PBMCs) was analyzed by flow cytometry using TB antigens (PPD), the polyclonal T cell activator staphylococcal enterotoxin B (SEB), or medium as unstimulated control.

**Results:**

Helminth infected CCs and LTBI subjects showed a significant reduction of IFN-γ^+^ CD4^+^ T cells by PPD-stimulation compared to non-helminth infected control groups. The significant reduction in the frequency of IFN-γ^+^ T cells in both latent and active PTB patients following SEB stimulation was mostly attributed to *Schistosoma mansoni* infection, whereas *Ascaris lumbricoides*, *Schistosoma mansoni*, and hookworm infection contributed equally in CCs. Following anti-helminthic and anti-TB treatment for 2 months, the frequency of IFN-γ^+^ CD4 T cells in helminth coinfected PTB was restored to levels of helminth negative PTB before treatment. Helminth coinfected PTB patients with an intermediate and severe clinical course had reduced capacity for production of IFN-γ^+^ T cells compared to the corresponding non-helminth infected PTB.

**Conclusion:**

We found a reduction in IFN-γ producing T cells by helminth coinfection which was restored following anti-helminthic treatment. This reduction was helminth species-dependent in an exploratory sub-analysis and correlated to increased disease severity.

## Author summary

Protective immunity against tuberculosis (TB) requires a Th-1 response with cytokines like TNF and IFN-γ which plays a key role in the recruitment and activation of immune cells. Helminth infection, on the other hand, can lead to induction of regulatory T cells and a Th-2 skewed response decreasing IFN-γ in T cells. Decreased Th-1 responses could favor reactivation of latent TB infection (LTBI), although the helminth species-specific effect on IFN-γ^+^CD4^+^ T cells and the link to TB disease severity in patients with active pulmonary TB (PTB) have not been fully investigated. Therefore, blood cells (PBMCs) from healthy controls, LTBI individuals, and PTB patients were used to evaluate the impact of different helminths on the frequency of IFN-γ^+^CD4^+^ T cells, in Gondar Ethiopia. *Ascaris lumbricoides*, *Schistosoma mansoni*, and hookworm infection in healthy controls contributed equally to decreasing the frequency of IFN-γ^+^CD4^+^ T cells, whereas in both LTBI and PTB patients *S*. *mansoni* coinfection had the greatest impact on reducing IFN-γ producing capacity of T cells. Decreased IFN-γ producing capacity of T cells was correlated with increased TB disease severity, only in helminth coinfected PTB patients, and anti-helminthic therapy restored the IFN-γ producing capacity of T cells at the 2 months follow-up.

## Introduction

Tuberculosis (TB) remains a major global public health problem killing 1.4 million people and causing over 10 million new cases per annum [1], and one fourth of the world population is latently infected with TB [2]. Similarly, helminthiasis affect over 1.5 billion people in the world [3]. There is a remarkable geographical overlap between TB and helminth infections and increased prevalence of helminths among TB patients compared to healthy community controls [4,5]. Helminth coinfection in TB patients has been shown to impair protective host immune responses [6]. In addition, helminth infection is suggested to increase the reactivation rate of latent TB infection (LTBI) and reduce the BCG vaccine response against TB [7].

Helminth infections induce a T helper-2 (Th-2) skewed immune responses characterized by interleukin 4 (IL-4), IL-5 and IL-13 cytokine responses and regulatory T cell responses with the production of IL-10 and tumor growth factor-β (TGF-β) [8]. In contrast, protective immunity against intracellular *Mycobacterium tuberculosis* (Mtb) requires a Th-1 dominated cytokine responses like interferon-gamma (IFN-γ), IL-2 and tumor necrosis factor (TNF) which plays a critical role in the pathogen killing mechanism [9].

Interferon-γ is a pleiotropic cytokine mainly produced by activated CD4^+^ T cells and CD8^+^ (cytotoxic) T cells [10], γδ T cells [11], and natural killer cells [12]. IFN-γ potentiates the pro-inflammatory signaling by priming monocytes and macrophages for anti-microbial actions since it initiates their production of microbiocidal reactive nitric oxide and oxygen intermediates and can stimulate the production of TNF which plays a key role in the killing process of intracellular Mtb [13]. IFN-γ also activates CD8^+^ T cells to produce IFN-γ, lyse Mtb infected macrophages, and killing of Mtb through granulysin [14]. Both IFN-γ knockout models in mice [15], as well as specific deficiencies in the IFN-γ pathway in humans, are associated with fatal disseminated mycobacterial infection [16]. In humans, IFN-γ gene polymorphisms are associated with an increased risk of LTBI [17] and the development of active TB [18]. Furthermore decreased IFN-γ was associated with severe TB disease [19], and increased IFN-γ production was associated with clinically cured TB [20]. A number of studies show that helminth infection modulates the protective Th-1 immune responses [21]. We have shown increased Th-2 cytokine profiles, and regulatory T cells in helminth TB coinfected individuals compared to patients with TB only and community controls [5,22]. There are limited clinical studies investigating the effect of helminth species-specific infection on the IFN-γ response of T cells in latent and active TB. Therefore, the aim of this study was to investigate the effect of helminth infection on IFN-γ producing capability of T cells during latent and active tuberculosis before and after treatment.

## Materials and methods

### Ethics statement

The University of Gondar ethical review board approved this study (O/V/P/RCS/05/1254/2016). All study participants gave their written informed consent prior to inclusion and they received appropriate care and treatment in accordance with Ethiopian national diagnosis and treatment guidelines. All biological specimens were collected and processed following appropriate standard operating procedures.

### Study participants

Consecutive pulmonary TB (PTB) patients were recruited from July 2016 to December 2018 from the Directly Observed Treatment Short course (DOTs) clinics of the University of Gondar Comprehensive Specialized Hospital and the Health Centers in Gondar, Maraki and Azezo Health Ethiopia. These four DOTs clinics, out of the seven available in Gondar, were selected based on patient flow, distance, and availability of immediate transportation of biological samples to the laboratory as the methodology required fresh specimens for processing with a maximum delay of two hours. HIV-negative PTB patients, confirmed by GeneXpert MTB/RIF assay, age 18–65 were included in the study. Patients with MDR/XDR TB, pregnancy, acute and chronic comorbidities or infection other than TB, and those requiring hospital admission were excluded. Healthy community controls (CCs) were recruited from blood donors at central Gondar Blood Bank and the rural community surrounding Gondar town, Ethiopia. Community controls (CCs) between the ages of 18–65, who were HIV-negative, had TB score values of ≤3 points and free from acute and chronic concomitant infection were included and classified as QFT-negative CCs. Additionally, CCs with a history of TB were excluded from the study. LTBI subjects were recruited among CCs and rural community control with a positive interferon-gamma release assay (QFT) test.

### Clinical examination

Socio-demographic and clinical data were collected from all participants using a structured questionnaire. The TB score value (a value from 0 to 13 points) was assessed using clinical parameters as previously described [23]; cough, chest pain, dyspnea, hemoptysis, night sweating, anemic conjunctivae, lung auscultation, tachycardia (≥100/min), raised temperature (≥37°C), body mass index (BMI) ≤18 kg/m2, BMI ≤16 kg/m2, mid-upper arm circumference (MUAC) ≤220 mm, and MUAC ≤200 mm and each parameter accounting for one point in the summed up TB score value. TB disease severity score were established according to the three severity classes: severity class I (SCI: 0–5 points), severity class II (SCII: 6–7 points), and severity class III (SCIII: 8–13), based on their TB score value [23].

### HIV screening

HIV screening was made by three rapid immune-chromatographic antibody tests for HIV1/2; HIV 1/2 STAT-PAK (Chembio Diagnostics systems Inc., USA), Uni-Gold Recombigen HIV-1/2 (Trinity Biotech, USA), and SD BIOLINE HIV-1/2 (Abbott, USA) at blood bank as the national routines for blood donor selection and DOTs clinics according to the provider-initiated HIV testing and counseling program (PIHCT). All test results were interpreted following the Ethiopian HIV/AIDS diagnosis and treatment guidelines. HIV-positive individuals were excluded from this study and linked to anti-retroviral therapy clinics for additional diagnosis, counseling, and treatment.

### Stool examination

Stool samples were collected from all study participants for the diagnosis of intestinal helminth infection. Stool microscopy was done using direct wet mount, formol-ether concentration and Kato-Katz techniques [24] following standard operating procedures by qualified laboratory technicians. Participants were categorized as helminth positive or helminth negative by combining all three technique results. One out of 10 was randomly assessed by another laboratory expert as part of a quality assurance system. Study participants with *S*. *mansoni* received Praziquantel with a calculated dose of 40 mg/kg in two divided doses. Similarly, subjects with *A*. *lumbricoides* and hookworm infection were treated with a single dose of 400 mg Albendazole.

### Sputum examination

In addition to clinical diagnosis of TB, pulmonary TB was determined from one spot sputum specimens from PTB patients by GeneXpert MTB/RIF cartridge-based nucleic acid amplification assay performed in accordance with the manufacturer’s instruction to diagnose pulmonary TB and detect rifampicin resistant strains of TB.

### Latent TB screening

Interferon-gamma release assay (QuantiFERON, QFT) test was used to screen for latent TB infection. Heparinised whole blood (1ml) was incubated in Nil (negative stimulation control), highly specific TB antigen (ESAT-6/CFP-10/TB-7.7 (p4)) and Mitogen (Positive stimulation control) containing tubes. Supernatants were harvested following 16–24 hr incubation at 37°C. The levels of released IFN-γ were measured using QuantiFERON-TB Gold ELISA kit (Qiagen, Australia) following manufacturers instruction and results was interpreted using QuantiFERON-TB Gold analysis software.

### Peripheral blood mononuclear cell (PBMC) isolation and cryopreservation

Ten ml of heparinized blood was collected and transported to the laboratory within 2 h of collection. The collected blood was diluted with an equal volume of phosphate-buffered saline (PBS) and layered on the LymphoPrep density gradient medium (Serumwerk, Bernburg AG, Oslo, Norway), and centrifuged at 800g for 30 min at 20°C without break. Following centrifugation, the PBMC-rich layer was harvested and washed twice with cold PBS using centrifugation at 200g for 10 min. The cells were suspended with 10% heat-inactivated fetal bovine serum (FBS) supplemented RPMI 1640 (Sigma-Aldrich, Munich, Germany) with 1% antibiotic antimycotic solution (RPMI-10), and counted via Bürker counting chamber using 0.4% trypan blue (Sigma-Aldrich, Munich, Germany) dye exclusion method. Isolated PBMCs were washed with RPMI-10 solution and stored with 10% dimethyl sulfoxide in FBS for less than 14 days at -80°C for flow cytometry analysis [25].

### *Ex vivo* PBMC stimulation and intracellular IFN-γ in T cells by flow cytometry

Cryopreserved PBMCs were thawed within 14 days of cryopreservation using pre-warmed RPMI-10 solution at 37°C and counted. PBMCs having >75% viability were used for experimental analysis. 5x10^5^ PBMCs in 500μl RPMI-10 were aliquoted into three tubes, one left unstimulated, one stimulated with 10μg/ml Mtb-derived purified protein derivative (PPD; Statens Serum Institute, Copenhagen, Denmark) to reveal Mtb-specific cytokine production, and 5μg/ml staphylococcal enterotoxin B (SEB, Sigma Aldrich, Germany) added to the third tube as a polyclonal T cell stimuli to serve as a positive control for a strong T cell cytokine response, and tubes incubated at 37°C for 2 h before 10μg/ml brefeldin A solution (Thermo Fisher Scientific, USA) was added to all tubes and reincubated at 37°C for another 4 h. Following incubation 5mM ethylene diamine tetra acetic acid (EDTA; Sigma-Aldrich, Munich, Germany) was added and incubated at 37°C for 15 min. Then PBMCs were washed with cold PBS and stained with extracellular staining antibodies (mouse anti-human antibodies: anti-CD3 (PerCPcy5.5, clone: UCHT1) and anti-CD4 (FITC, clone: RPA-T4) both from BD Bioscience, USA) at room temperature in the dark for 30 min. Then PBMCs were washed with cold PBS and fixed/permeabilized for 20 min at 4°C with cytofix/cytoperm solution (BD Bioscience, USA). PBMCs were washed using 1xpermwash solution (BD Bioscience, USA) and stained with intercellular staining using the mouse anti-human antibody: anti-IFN-γ (Alexa Fluor 647, clone: B27) (BD Bioscience, USA) at room temperature in dark for 30 min. Stained PBMCs were washed twice with 1xpermwash solution and fixed with 1% paraformaldehyde solution. Flow cytometer acquisition of stained PBMCs was performed by a FACS Calibur flow cytometer (BD Biosciences) using Cell Quest acquisition software, and raw data analysis performed using FlowJo 7.6.5 software (Tree Star, USA).

### Statistical analysis

Descriptive statistics were used to describe socio-demographic and clinical characteristics of study participants. Continuous data are presented as mean ± SEM and Graph pad prism v.5 statistical software used for statistical analysis. Unpaired two-sided Student’s t-test was used to compare the differences between helminth negative and combined helminth positive QFT^-^CCs, LTBI and TB positive groups. One-way ANOVA followed by Tukey’s multiple comparison test was used to assess helminth species-specific effects within each group. Two-way ANOVA followed by Bonferroni post-test was applied to determine differences between the data obtained from follow-up periods and for comparison of different disease severity classes of helminth negative and positive TB patients. We estimated that 9 patients in the helminth negative and helminth positive groups respectively (TB patients, latent TB patients and CCs) were needed to show a reduction of IFN-γ^+^ CD4^+^ T cells from 6.5% (assuming a standard deviation of 1.5) to 4.5% of all CD4^+^ T cells with a power of 80% and an alpha of 0.05.

## Results

### Study participants and clinical characteristics

**Fig 1:**
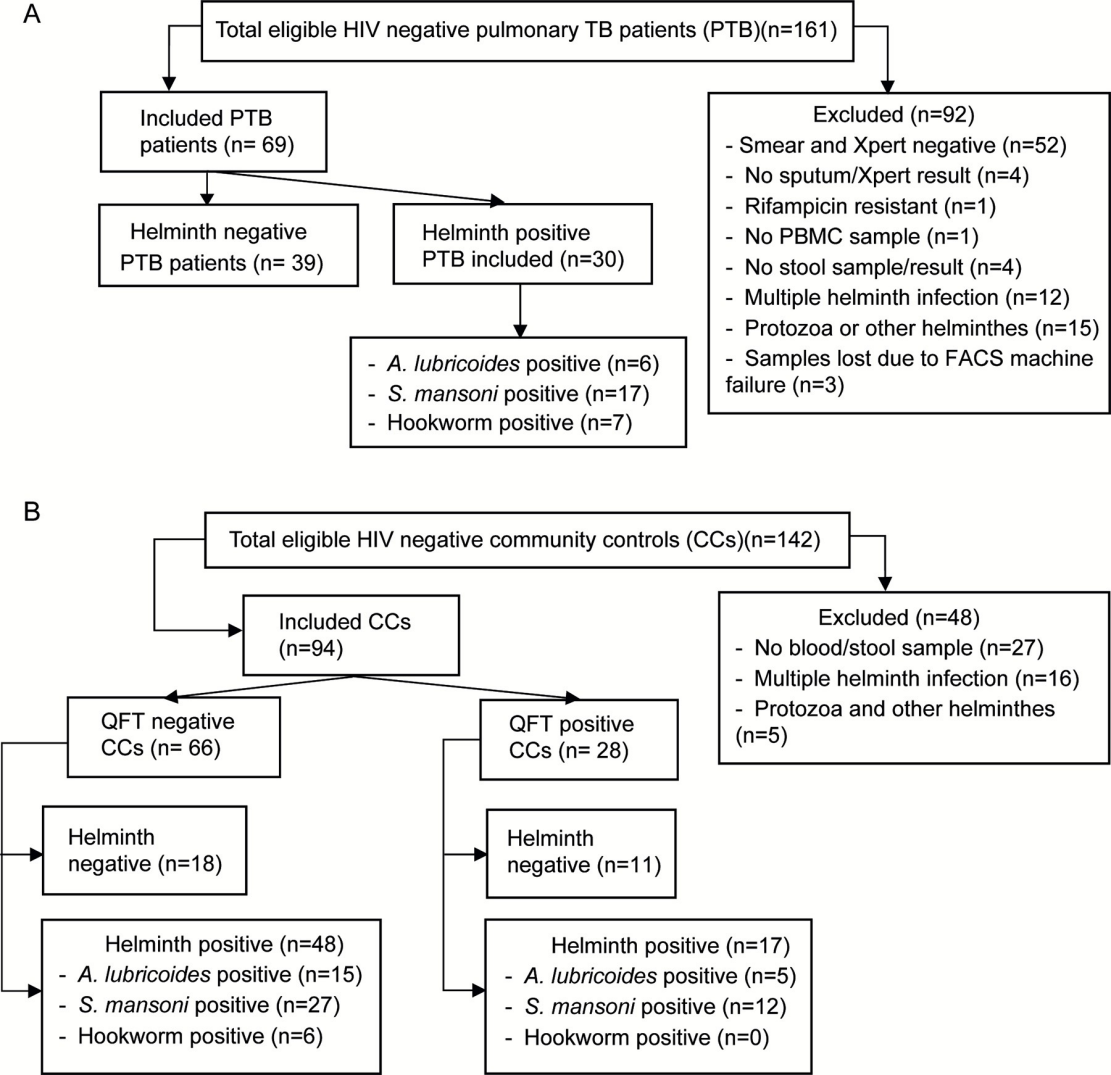
Study participant recruitment chart.

A total of 163 study participants were included in this study, of these 66 (40.5%) were QuantiFERON negative healthy community controls (QFT^-^ CCs), 28 (17.2%) were QuantiFERON positive subjects with latent TB (QFT^+^CCs; LTBI), and 69 (42.3%) were newly diagnosed active pulmonary tuberculosis patients. The TB patients were Xpert MTB/RIF confirmed cases out of 161 eligible subjects with a clinical suspicion of TB (Fig 1). Among TB patients included in the study, 43.4% (30/69) were helminth positive, similar to what has been previously reported in the area [5,26]. The distribution of helminth species in these groups along with baseline data are described in Table 1.

**Table 1:** Clinical characteristics of study participants.

Groups	Helminth	Sex n(%)	Age	BMI	TB score
N (%)	Status, n(%)	Male, Female	Mean ± SD	Mean ± SD	Mean ± SD
QFT^-^ CCs 66 (40.5%)	Helminth neg., 18(27.3)	13(72.2), 5(27.8)	26.1±3.1	22.2±3.4	0.2±0.7
	*A*.*lumbricoides*^+^, 15(22.7)	5(33.3), 10(66.6)	32.6±11.3	20.1±4.1	1.4±1.3
	*S*.*mansoni*^+^, 27(40.9)	17(63), 10(37)	25.3±8.7	21.0±3.4	1.0±1.2
	Hookworm^+^, 6(9.1)	3(50), 3(50)	23.2±5.0	20.8±1.7	0.2±0.4
p value for QFT^-^ CCs*			NS	NS	NS
QFT^+^ CCs; LTBI; 28 (17.2%)	Helminth neg., 11 (39.3)	10(90.9), 1(9.1)	28.6±5.1	22.5±3.5	0.7±1.1
	*A*.*lumbricoides*^+^, 5(17.9)	2(40), 3(60)	40.2±14.1	20.6±1.0	1.2±1.1
	*S*.*mansoni*^+^, 12(42.8)	7(58.3), 5(42.7)	38.2±13.7	19.5±1.9	1.5±1.3
p value for LTBI			NS	NS	NS
PTB patients 69 (42.3%)	Helminth neg., 39(56.5)	13(33.3), 16(66.6)	29.3±12.6	18.1±1.5	6.6±2.3
	*A*.*lumbricoides*^+^, 6(8.7)	5 (83.3), 1(16.7)	25.6±9.2	17.8±1.8	6.3±1.9
	*S*.*mansoni*^+^, 17(24.6)	12(70.6), 5 (29.4)	27.1±9.3	18.2±2.2	6.4±2.1
	Hookworm^+^, 7(10.1)	5(71.4), 2(28.6)	39.1±17	17.8±1.4	5.7±2.4
p value for PTB			NS	NS	NS

N, number; neg., negative; BMI, body mass index; QFT, QuantiFERON test; PTB, pulmonary tuberculosis; CCs, community controls; LTBI, latent tuberculosis infection; NS, non-significant.

*The p-value represents comparison of means using one way ANOVA between helminth negative, *A*.*lumbricoides*^+^, *S*.*mansoni*^+^ and hookworm^+^ groups of QFT^-^CCs, LTBI and PTB groups.

### Helminth infection is associated with impaired T cell IFN-γ production in LTBI-negative healthy community controls

**Fig 2:**
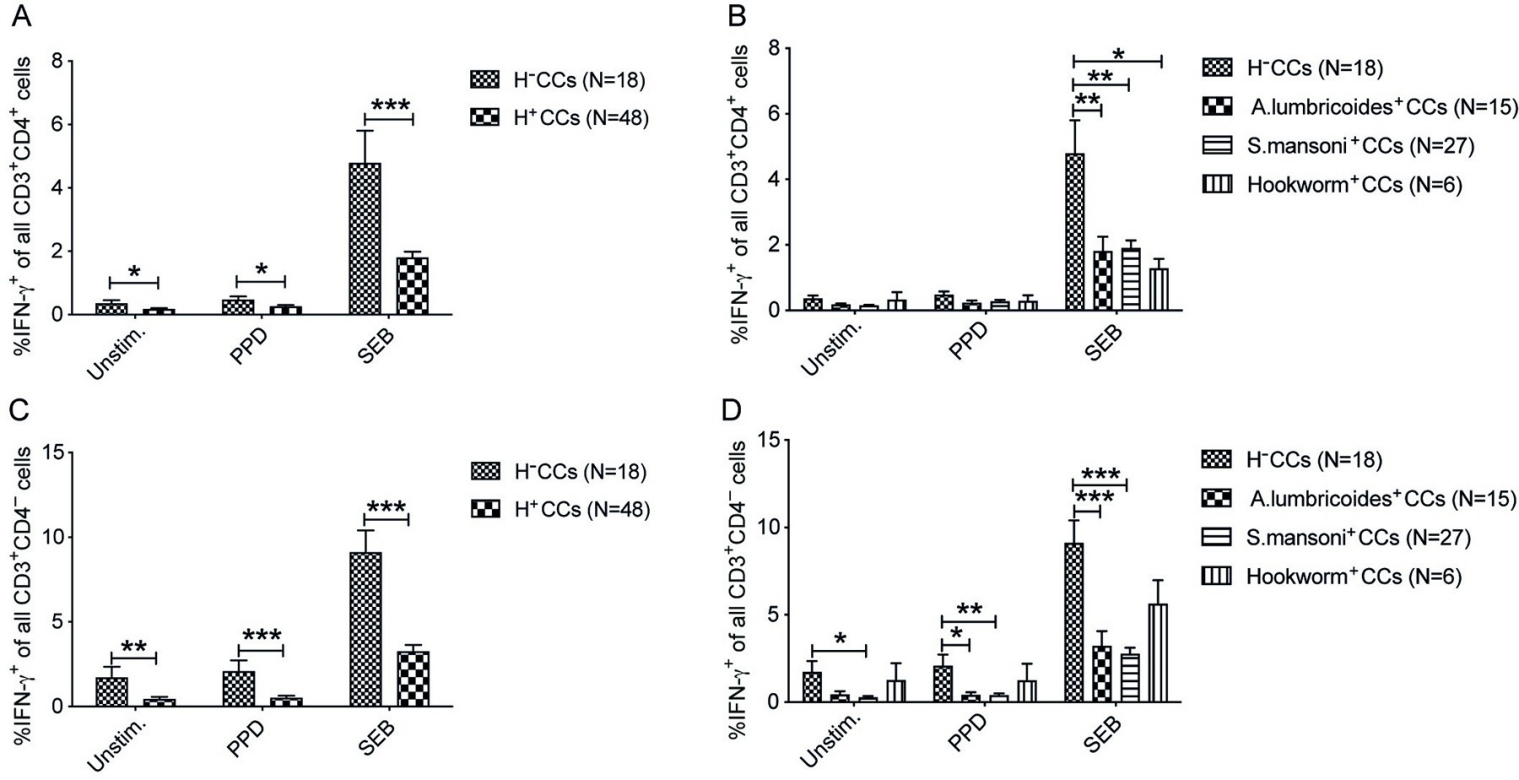
Helminth infection decreases IFN-γ producing capacity of T cells in QFT^-^CCs. PBMCs from community controls (QFT^-^CCs) with and without helminth infection were *ex vivo* stimulated with PPD, SEB, or left unstimulated (Unstim.) and incubated with Brefeldin A for flow cytometry analysis of intracellular IFN-γ expression in CD3^+^ T cells. Frequency of IFN-γ^+^CD4^+^ T cells of all CD3^+^CD4^+^ cells in helminth negative (H^-^CCs) and combined helminth positive (H^+^CCs) (A), H^-^ and specific helminth infection (*A*. *lumbricoides*^+^, *S*. *mansoni*^*+*^ and hookworm^+^) (B). Frequency of IFN-γ^+^CD4^-^ T cells of all CD3^+^CD4^-^ cells in H^-^CCs versus H^+^CCs (C) and H^-^CCs versus specific helminth infection (D). Data presented as mean ± SEM and the unpaired two-sided Student’s t-test was used to analyze the differences between H^-^ and combined H^+^ CCs, whereas one-way ANOVA following Tukey’s multiple comparison test was used to test the species-specific effect. *, p < 0.05; **, p < 0.01; ***, p < 0.001.

In this study, we analyzed the frequency of T cells (CD3-positive cells) in PBMCs, either CD3^+^CD4^+^ or CD3^+^CD4^-^, that *ex vivo* were positive for intracellular IFN-γ, hereafter expressed as IFN-γ^+^CD4^+^ T cells and IFN-γ^+^CD4^-^ T cells, respectively. Helminth infected QFT^-^CC subjects had a significantly lower frequency of IFN-γ^+^CD4^+^ T cells in unstimulated (p<0.05), PPD (p<0.05), and SEB (p<0.001) stimulated PBMCs compared to helminth negative QFT^-^CCs (Fig 2A). Similarly, the frequency of IFN-γ^+^CD4^-^ T cells was significantly lower in unstimulated (p<0.001), PPD (p<0.001), and SEB stimulated PBMCs (p<0.001) of helminth infected QFT^-^CCs compared to helminth negative QFT^-^CCs (Fig 2C). The helminth species-specific data analysis showed a lower frequency of IFN-γ^+^CD4^+^ T cells in PBMCs of *A*. *lumbricoides* (p<0.001), *S*. *mansoni* (p<0.001), and hookworm (p<0.05) infected QFT^-^CCs compared to helminth negative QFT^-^CCs when stimulated with SEB, whereas unstimulated and PPD stimulation did not show any differences (Fig 2B). Additionally, *A*. *lumbricoid*es infected CCs had decreased frequency of IFN-γ^+^CD4^-^ T cells in PPD (p<0.05) and SEB stimulation (p<0.001), whereas in *S*. *mansoni* infected CCs there was a lower frequency of IFN-γ^+^CD4^-^ T cells in unstimulated (p<0.05), PPD (p<0.001) and SEB (p<0.001) stimulated compared to helminth negative QFT^-^CCs (Fig 2D).

### *S*. *mansoni* infection reduces IFN-γ positive T cells in PBMCs of subjects with latent TB infection

**Fig 3:**
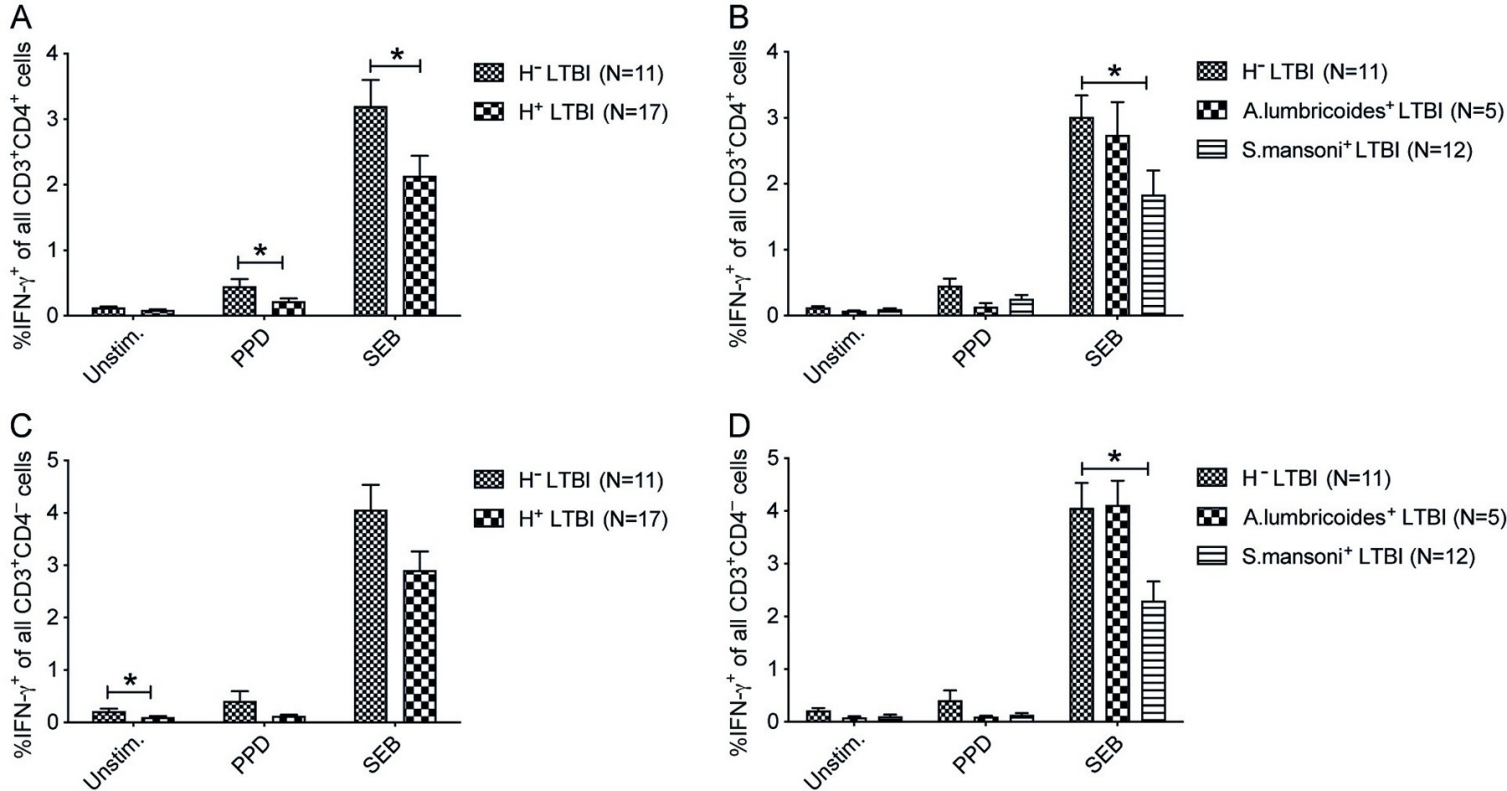
*S*. *mansoni* infection suppresses IFN-γ producing capacity by CD3^+^ T cells in LTBI positive individuals. PBMCs from helminth negative and helminth positive LTBI individuals were *ex vivo* stimulated with PPD, SEB, or left unstimulated (Unstim.) and incubated with Brefeldin A for analysis of IFN-γ producing T cell by flow cytometry. Frequency of IFN-γ^+^CD4^+^ cells of all CD3^+^CD4^+^ cells in helminth negative (H^-^LTBI) and combined *A*. *lumbricoides* and *S*. *mansoni* positive (H^+^LTBI) (A), H^-^ versus specific helminth infection (B). Frequency of IFN-γ^+^CD4^-^ cells of all CD3^+^CD4^-^ cells in H^-^LTBI versus H^+^LTBI (C), and H^-^LTBI versus specific helminth infection (D). Data presented as mean ± SEM, and Student’s t-test was used to analyze the differences between H^-^LTBI and H^+^LTBI whereas one-way ANOVA following Tukey’s multiple comparison test was used to assess the species-specific effect. *, p < 0.05.

LTBI positive individuals (QFT^+^) with helminth infection showed a lower frequency of IFN-γ^+^CD4^+^ T cells in PPD (p< 0.05) and SEB (p< 0.05) stimulation (Fig 3A), as well as IFN-γ^+^CD4^-^ T cells with a similar pattern (Fig 3C), when compared to helminth negative LTBI subjects. Analyzing species specific helminths, *S*. *mansoni* infected subjects showed a reduced frequency of IFN-γ^+^CD4^+^ T cells (p<0.05) and IFN-γ^+^CD4^-^ T cells (p<0.05) in SEB stimulated PBMCs, whereas *A*. *lumbricoides* infection did not show any influence on the frequency of IFN-γ^+^CD4^+^ or IFN-γ^+^CD4^-^ T cells (Fig 3B and 3D).

### *S*. *mansoni* coinfection in active pulmonary TB is associated with a profound reduction in IFN-γ production of CD4^+^ T cells

**Fig 4:**
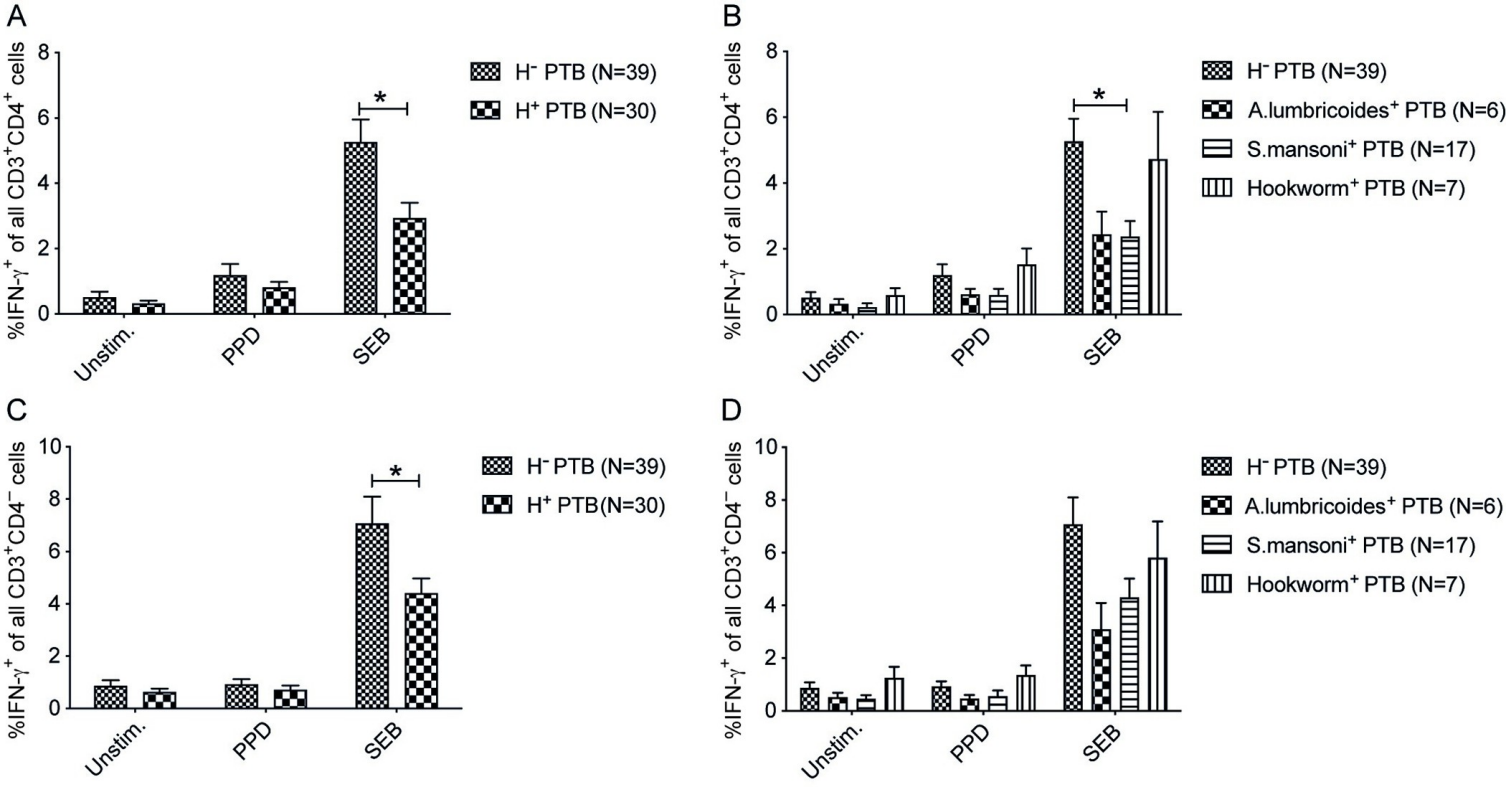
Decreased IFN-γ production by CD3^+^ T cells in pulmonary TB patients with *S*. *mansoni* coinfection. Unstimulated, PPD and SEB stimulated PBMCs of PTB patients were incubated with Brefeldin A *ex vivo* to analyze intracellular IFN-γ expression in CD3^+^CD4^+^ and CD3^+^CD4^-^ T cells by flow cytometry. Frequency of IFN-γ^+^CD4^+^ T cells in helminth negative PTB (H^-^PTB) and combined *A*. *lumbricoides*, *S*. *mansoni* and worm positive PTB patients (H^+^PTB) (A), and H^-^PTB versus specific helminth infection (B). Frequency of IFN-γ^+^CD4^-^ T cells of all CD3^+^CD4^-^ cells in H^-^PTB and combined H^+^PTB (C), and specific helminths in PTB patients (D). Data presented as mean ± SEM and the unpaired two-sided Student’s t-test was used to analyze the differences between H^-^PTB and H^+^PTB whereas one-way ANOVA followed by Tukey’s multiple comparison test was used to assess the species specific effects. *, p < 0.05.

Analysis performed when all helminths were combined in one group showed that helminth-infected PTB patients had a decreased frequency of IFN-γ^+^CD4^+^ T cells (p<0.05) (Fig 4A) and IFN-γ^+^CD4^-^ T cells (p<0.05) (Fig 4C) in SEB stimulated PBMCs compared to helminth negative PTB patients. The helminth species-specific analysis revealed that *S*. *mansoni* infection resulted in a significant reduction of IFN-γ^+^CD4^+^ T cell frequency (p<0.05) in SEB stimulated PBMCs. Further, *A*. *lumbricoides* showed a non-significant reducing trend of IFN-γ^+^CD4^+^ T cells while hookworm infection had little effect. These results suggested that the effect of helminth infection in reducing IFN-γ production varies with different helminth species.

### Anti-helminthic treatment significantly increased IFN-γ production in CD4^+^ T cells of helminth-positive PTB patients at the 2-month follow-up

**Fig 5:**
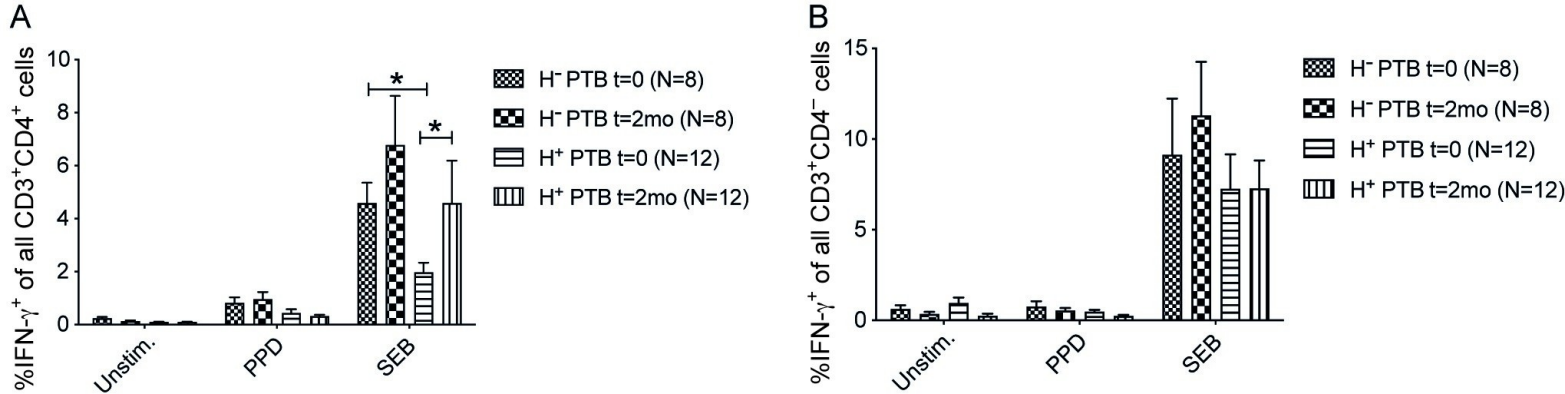
IFN-γ producing capacity of CD4^+^ T cells enhanced in helminth positive PTB at the two months of TB and anti-helminthic treatment follow-up. Anti-TB drugs were administered to all PTB patients and helminth positive patients (H^+^PTB) received additional anti-helminthic treatment. Unstimulated, PPD and SEB stimulated PBMCs were *ex vivo* incubated with Brefeldin A to analyze intracellular IFN-γ expression in CD3^+^CD4^+^ and CD3^+^CD4^-^ T cells by flow cytometry. Frequency of IFN-γ^+^CD4^+^ T cells of all CD3^+^CD4^+^ cells (A) and frequency of IFN-γ^+^CD4^-^ T cells of all CD3^+^CD4^-^ cells (B) in helminth negative PTB (H^-^PTB) and combined (H^+^PTB) PTB patients. Data presented as mean ± SEM, and two-way ANOVA with Bonferroni posttest used to assess differences between groups. *, p < 0.05.

Before anti-TB and anti-helminth treatment of the helminth positive PTB patients (t = 0), helminth positive PTB patients had a significantly lower frequency of IFN-γ^+^CD4^+^ T cells compared to helminth negative PTB patients (p < 0.05) in SEB stimulated PBMCs, which is the stimuli here used to induce a total or maximum T cell cytokine production. At 2-months of treatment follow-up, the difference in IFN-γ^+^CD4^+^ T cells among helminth positive was not significantly different from that in helminth negative at t = 0, with SEB stimulation. A comparison before (t = 0) and after 2 months of treatment for the SEB-induced frequency of IFN-γ^+^CD4^+^ T cells within each group showed a significant increase at 2 months in helminth positive PTB patients (p <0.05) also receiving anti-helminthic treatment (Fig 5 A). Sub-analysis with regards to specific helminth species was not possible due to the limited number of patients available for follow-up. However, it turned out that *S*. *mansoni* was the dominant helminth species among the helminth positive in this follow-up group, either as *S*. *mansoni* single-helminth infected (n = 7) or multiple helminth-infected including *S*. *mansoni* (n = 5), all treated with praziquantel for Schistosomiasis.

### Reduced IFN-γ production of T cells in helminth positive PTB patients with intermediate and severe clinical scores compared to mild clinical TB cases

**Fig 6:**
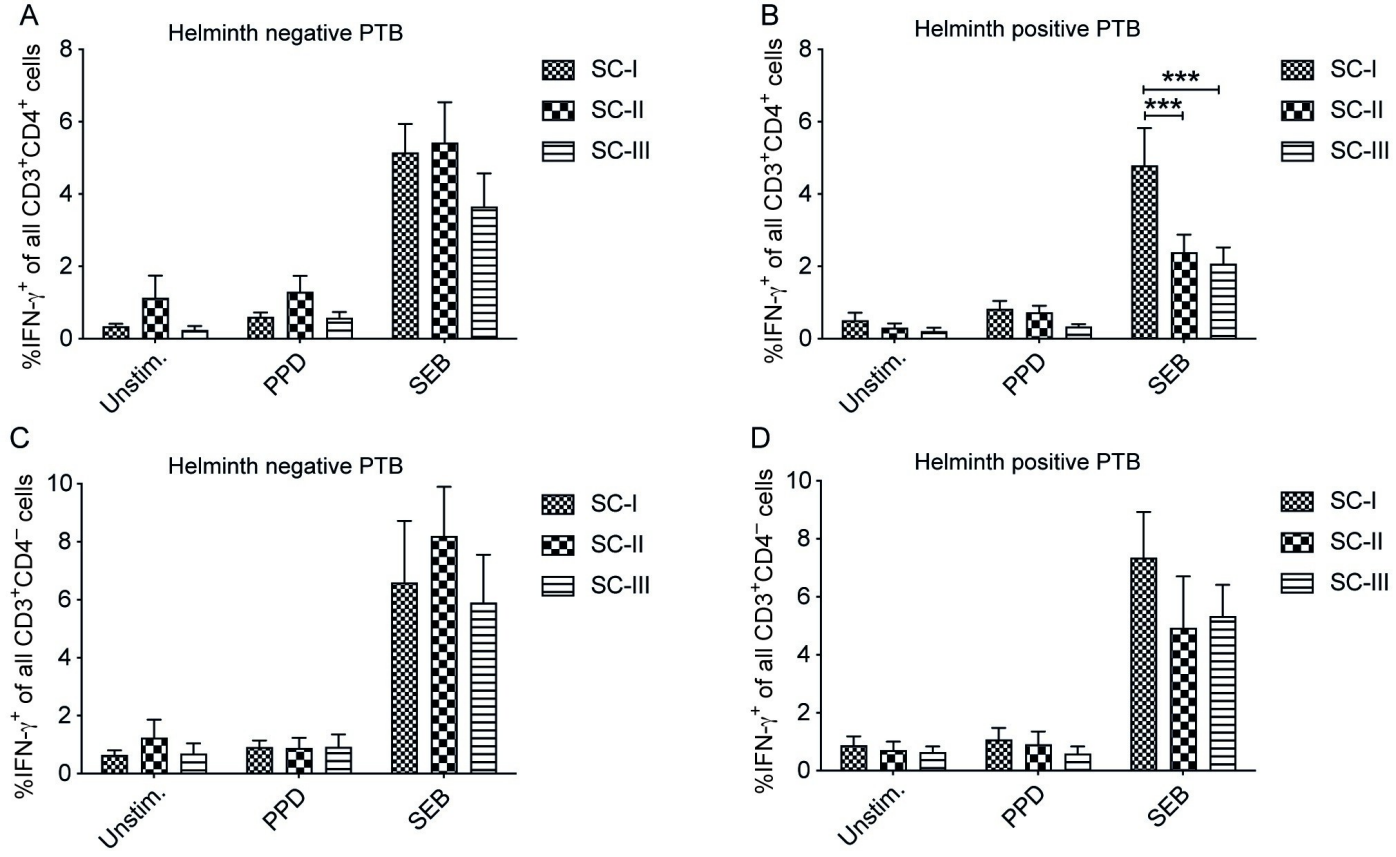
Reduced capacity for IFN-γ production in CD4^+^ T cells of helminth infected PTB patients having intermediate and sever clinical course of TB. Unstimulated, PPD and SEB stimulated PBMCs were incubated with Brefeldin A *ex vivo* to analyze intracellular IFN-γ expression in CD3^+^CD4^+^ and CD3^+^CD4^-^ T cells by flow cytometry. Frequency of IFN-γ^+^CD4^+^ T cells of all CD3^+^CD4^+^ cells in helminth negative PTB patients (A) and combined *A*. *lumbricoides*^+^, *S*. *mansoni*^+^ and hookworm^+^ PTB patients (helminth positive PTB) (B). The frequency of IFN-γ^+^CD4^-^ T cells of all CD3^+^CD4^-^ T cells in helminth negative PTB patients (C) and helminth positive PTB patients (D). Data presented as mean ± SEM in three disease severity classes (SCI, n = 14/10; SCII, n = 10/10; and SCIII, n = 13/10 in helminth negative/positive PTB patients respectively). Two-way ANOVA with Bonferroni posttest was used to compare differences between different severity classes of H^-^PTB and H^+^PTB patients. ***, p < 0.001.

Analysis to explore the connection between IFN-γ producing capacity of T cells and disease severity as classified into three TB severity classes (SCI-III) using TB score showed a significantly lower frequency in SEB stimulated PBMCs of helminth positive PTB patients with SCII (p < 0.001) and SCIII (p < 0.001) compared to helminth positive PTB patients of the SCI group (Fig 6B). Comparison between helminth positive and helminth negative PTB groups of the same TB disease severity class showed a lower frequency of IFN-γ^+^CD4^+^ T cells only in helminth positive PTB patients with SCII (p < 0.05) compared to helminth negative PTB patients with SCII. In this analysis, subgrouping of data with regards to disease severity resulted in too few patients per subgroup to perform an analysis with regards to helminth species.

## Discussion

In this study, the main finding in the context of intracellular cytokine production of CD4^+^ T cells is that helminth infection reduced the frequency of IFN-γ^+^ T cells in a helminth species dependent pattern. Before treatment, high disease severity was linked to a reduction in the IFN-γ^+^ T cell response in helminth coinfected patients where anti-helminth and TB treatment restored the IFN-γ producing capacity by T cells after two months.

Helminth infection significantly suppressed the expression of IFN-γ by T cells in active, latent TB and also in QFT-negative CCs. As a novel strategy compared to previous studies, the species-specific effect of Ascaris, *S*. *mansoni* and hookworm was investigated in the present study. We found that the capacity to produce IFN-γ by T cells varied according to the helminth species. In QFT-negative CCs, helminth infection resulted in decreased frequency of IFN-γ^+^CD4^+^ T cells in unstimulated, PPD as well as SEB stimulated PBMCs compared to helminth negative QFT-negative CCs, and Ascaris, *S*. *mansoni*, and hookworm infections all showed a similar impact with more than a 50% reduction. This is consistent with previous studies showing a significantly decreased IFN-γ production from PBMCs of chronic *S*. *mansoni* infected individuals [27] and mitogen-induced IFN-γ from PBMCs of Ascaris infected individuals [28]. This suppressed IFN-γ producing capacity of T cells might be related to the mechanism whereby helminth infections induce a Th-2 skewed immunity and increase in regulatory T cell responses for the establishment of long-standing infection in the host [29] which then greatly contribute to established infections by intracellular pathogens such as Mtb where a suppressed immune response enhances their risk for disease progression.

In LTBI positive individuals combined helminth infection reduced IFN-γ^+^CD4^+^ cells in PBMCs stimulated with PPD and SEB. *S*. *mansoni* infection decreased IFN-γ^+^CD4^+^ T cells but Ascaris infection had little to no effect. This profound suppression in Mtb-specific and maximum IFN-γ production by T cells in helminth positive individuals may be due to the expansion of Mtb-specific memory cells primed to produce Th-2 type cytokines [30], or CD4^+^ T cell-related epigenetic changes induced by helminth infection as previously described for Schistosomiasis and Ascaris infection in recently TB-exposed children where CD4^+^ T cell DNA hypermethylation resulted in decreased TB-specific IFN-γ and TNF expression [31]. Our results on LTBI individuals are also consistent with a study showing a decreased frequency of PPD-induced IFN-γ^+^CD4^+^ cells in helminth positive LTBI individuals, compared to helminth negative LTBI individuals, which was reversed following anti-helminthic therapy [32]. This suppressed immune response may increase the risk of reactivating TB.

In active TB patients, we found a decreased frequency of IFN-γ^+^CD4^+^ T cells in helminth positive TB patients which is consistent with previous studies that showed diminished systemic Th-1 and Th-17 cytokine responses [33] and mycobacterial-specific mono- and poly-functional Th-1 and Th-17 cells during helminth coinfection [34]. A lower IFN-γ may lead to poor activation of macrophages which will reduce the capacity to kill intracellular Mtb, as previously described for PBMCs exposed with schistosomal egg antigen resulting in a decreased frequency of TB-specific IFN-γ^+^CD4^+^ cells and poor Mtb control by macrophages [35]. Similarly, in TB patients we observed a significantly reduced frequency of IFN-γ^+^CD4^+^ T cells with *S*. *mansoni* and Ascaris showed a non-significant 2-fold reduction, whereas hookworm coinfection did not affect the frequency of IFN-γ^+^CD4^+^ T cells to the same extent, although these results should be interpreted with caution due to the few subjects included. These results indicate the variable effects of helminths in the suppression of immune response against TB, and we have recently revealed a helminth species-dependent increase in TGF-beta positive functionally active regulatory T cells [22] and helminth species-dependent expansion of non-classical monocytes in TB patients [36].

Helminth positive active PTB patients had a lower frequency of IFN-γ^+^CD4^+^ T cells at inclusion and this was reversed following treatment with anti-helminthic drugs showing that deworming can improve the IFN-γ producing capacity of CD4^+^ T cells at the 2-month follow-up compared to t = 0. There is a scarcity of data showing the effect of deworming on IFN-γ production by T cells in active TB patients. In our previous work, we showed that Albendazole treatment of coinfected TB patients decreases the frequency of eosinophils and the IL-10 response [37]. Additionally, LTBI individuals with *Strongyloides stercoralis* and *S*. *mansoni* had a decreased frequency of IFN-γ^+^CD4^+^ T cells [32] and lower Th-1 cytokine responses to TB-antigen stimulation during Strongyloides infection that was reversed following anti-helminthic therapy [38]. This suggests that anti-helminthic treatments during coinfection improve the immunological response which might, in turn, enhance the clinical outcome of the patient although this needs to be confirmed in prospective clinical studies.

We found an overall correlation between severe TB disease and an impaired IFN-γ response as previously shown [39]. In relation to helminth coinfection, based on the TB score, the intermediate and severe clinical classes of TB patients had a significantly lower frequency of IFN-γ^+^CD4^+^ T cells in PBMCs of helminth positive TB patients compared to helminth positive TB patients with SCI. Our finding is consistent with that of helminth coinfected TB patients showing decreased IFN-γ in whole blood culture supernatants that were associated with severe radiological pulmonary disease showing multiple involved lung zones at the end of TB treatment [40].

Our study has several limitations. First, even if we have a relatively large total sample size for *ex vivo* stimulation of PBMCs, there are few subjects in each subgroup of helminths and in particular for hookworm. There is a need for confirmatory studies to verify the findings of this exploratory sub analysis. Secondly, we did not further confirm our flow cytometry based intracellular IFN-γ results using alternative methods. Thirdly, we did not perform a follow-up data analysis specific to helminth species. Presently, the follow-up data of dewormed PTB patients are most indicative of the response among PTB patients with *Schistosoma mansoni*, as all helminth positive in this group had at least a *Schistosoma mansoni* infection and the 2-month follow-up response was measured after this group was treated with praziquantel for Schistosomiasis.

In summary, our results show a reduced T cell IFN-γ response in helminth infected CCs, LTBI, and active pulmonary TB patients compared to the corresponding groups without helminth infection. In an exploratory sub analysis, IFN-γ responses were dependent on helminth species and impaired in severe TB disease but reversed following anti-helminthic treatment. The public health implications of these findings for helminth and TB coinfection may be a higher risk for LTBI subjects with helminth infection to reactivate into active TB due to a reduced IFN-γ mediated immune control. The clinical implications of these findings should be investigated in a multicenter prospective study to evaluate if treatment of helminth infection in patients with LTBI is warranted on a routine basis.
